# Comparison of the perioperative parameters between computer navigation and fluoroscopy guidance for pedicle screw placement: A protocol for a systematic review and meta-analysis

**DOI:** 10.1097/MD.0000000000021064

**Published:** 2020-07-10

**Authors:** Qianchun Li, Botao Chen, Rigao Chen, Yang Yu, Leiming Jiang, Xiaohong Fan

**Affiliations:** Department of Orthopedics, Hospital of Chengdu University of Traditional Chinese Medicine, Chengdu, Sichuan Province, China.

**Keywords:** fluoroscopy, meta-analysis, navigation, pedicle screws, protocol, spine, surgery

## Abstract

**Background:**

Computer navigation technology is gradually applied to the placement of pedicle screws, but its security and effectiveness still lack of high-quality evidence-based medical evidence. In this study, we will perform a systematic review of previously published randomized controlled trials to investigate the accuracy and effectiveness of computer navigation vsersus fluoroscopy guidance for pedicle screw placement.

**Methods:**

All study protocols adhered to the Preferred Reporting Items for Systematic Reviews and Meta-Analyses guidelines. PubMed (MEDLINE), The excerpta medica database, Web of Science (science and social science citation index), The Cochrane Library (Cochrane Database of Systematic Reviews, Cochrane Central Register of Controlled Trials (CENTRAL), Cochrane Methodology Register), China National Knowledge Infrastructure, Chinese Science and Technology Periodical Database, WanFang, Chinese Biomedical Literature Database will be searched for relevant articles up to 18 April, 2020. We will include randomized controlled trials of computer navigation and fluoroscopy guidance for pedicle screw placement. The Cochrane Handbook (v6) will be used for assessment of study bias and reliability, and a meta-analysis will be performed using STATA 16.0. The main outcome will be the proportion of accurate implanted screws. Additional outcomes including: overall complication rate, radiation dosage, length of surgery, length of stay, estimated blood loss.

**Results:**

The quality of the assessments will be assessed through Grading of Recommendations Assessment, Development, and Evaluation. Data will be disseminated through publications in peer-reviewed journals.

**Conclusion:**

We will evaluate the accuracy and other perioperative parameters between computer navigation and fluoroscopy guidance for pedicle screw placement.

**Trial registration number:**

PROSPERO 2020 CRD42020172087.

## 1 Introduction

In 1959, Boucher^[1]^ first reported the pedicle screw (PS) fixation technique. After Harrington^[2]^, Roy Camille^[3]^ and other improvements, it is widely used in spine corrective, fixation and fusion operations, and is now 1 of the most frequently performed spinal surgery procedures. PSs have good biomechanical properties, which can bring favorable results in the treatment of various spinal diseases.

Conventionally, PS implantations are mainly assisted by anatomic landmarks and intraoperative C-arm fluoroscopy.^[4]^ There is a high rate of misalignment, even for experienced spine surgeons. It was previously shown that 2.39% to 10% of patients required revision surgery when percutaneous PSs implantation.^[5–8]^ In the open surgery, 10% to 40% cases were reported dislocated, and the rate of revision was as high as 7.6%.^[9,10]^ In addition, more and more attention has been paid to the risk of radiation exposure of doctors and patients caused by intraoperative fluoroscopy. From the biomechanical point of view, PSs should be ideally placed along the pedicle axis to ensure the appropriate screw diameter and length, and the ideal position also ensures the best protection of adjacent structures. PS dislocation can lead to serious complications, such as involve potential nerves, vital blood vessels, and Viscera damage to structures neighboring the spine.^[11]^ If the screw position is found to be undesirable through fluoroscopy during the operation, it can be adjusted in time, but repeatedly regulate the screw track will affect the holding force and lead to failure of internal fixation, which will affect the stability and fusion of the spine. What's worse is that only after the operation, the dislocation of screw position is found with complications, and it needs to be renovated, which will seriously affect clinical efficacy, patient satisfaction, and medical expenses. Therefore, the initial accuracy of PS is very critical.

In order to improve the safety and accuracy, navigation and robot technology are applied to the PS implantation, especially in the percutaneous PS technology.^[12–14]^ A series of different navigation systems have been used in PS insertion since 1995^[15]^, and even more (especially in the field of Robotics since 2006^[16]^) are under development or just beginning to be adopted^[17–23]^.

At present, there are few randomized controlled studies on robots. In this study, we will systematically review published randomized controlled trials (RCTs) and perform a meta-analysis comparing computer navigation vs. fluoroscopy guidance to evaluate the accuracy of screw placement and other related perioperative indicators, such as overall complication rate, radiation dosage, length of surgery, length of stay, and estimated blood loss. Which could provide relevant data support for the development of digital orthopedics, evaluate the advantages and disadvantages of current navigation technology, and provide relevant support for the improvement or research and development of new navigation system and robot system.

## 2 Methods

### 2.1 Design and registration of the Study

All methods will conform to Preferred Reporting Items for Systematic Reviews and Meta-Analyses (PRISMA) protocols guidance ^[24,25]^ and has been registered in PROSPERO 2020 (CRD42020172087). All data analysis will adhere to the PRISMA statement.^[26,27]^

### 2.2 Eligibility criteria

#### 2.2.1 Study type

Prospective RCTs that compare the accuracy and other perioperative parameters between computer navigation and fluoroscopy guidance for PS placement.

#### 2.2.2 Participants

Adults (≥18 years) that underwent posterior PS fixation of the spine

#### 2.2.3 Types of interventions

Computer navigated surgery.

#### 2.2.4 Comparison

Fluoroscopy guidance (conventional).

#### 2.2.5 Study parameters

##### 2.2.5.1 Primary outcomes

(1) Proportion of accurately implanted screws according to 0 mm grading criteria
(2) Proportion of accurately implanted screws according to 2 mm grading criteria

##### 2.2.5.2 Secondary outcomes

(1) Overall complication rate
(2) Radiation dosage
(3) Length of surgery
(4) Length of stay
(5) Estimated blood loss

#### 2.2.6 Exclusion criteria

Republished literature, unable to obtain full text or data, the type of study could not be confirmed and fundamental research will be excluded.

### 2.3 Search strategy

All the major electronic databases will be searched from inceptions to the April 18, 2020. The basic search strategy is outlined in Table 1.

**Table 1:**
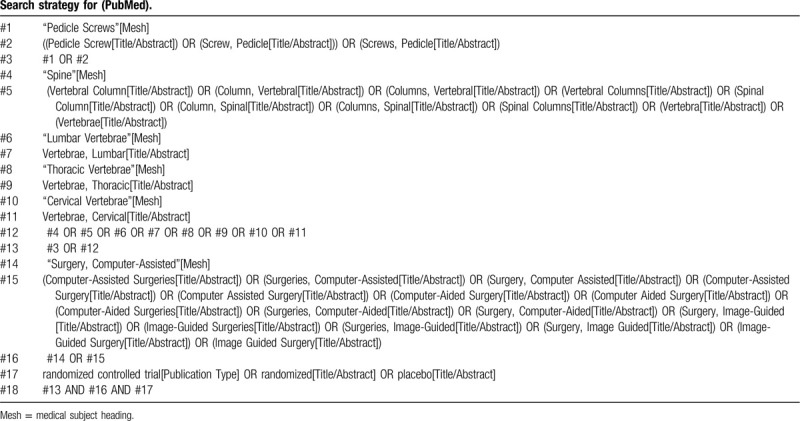
Search strategy for (PubMed).

### 2.4 Study selection

**Figure 1:**
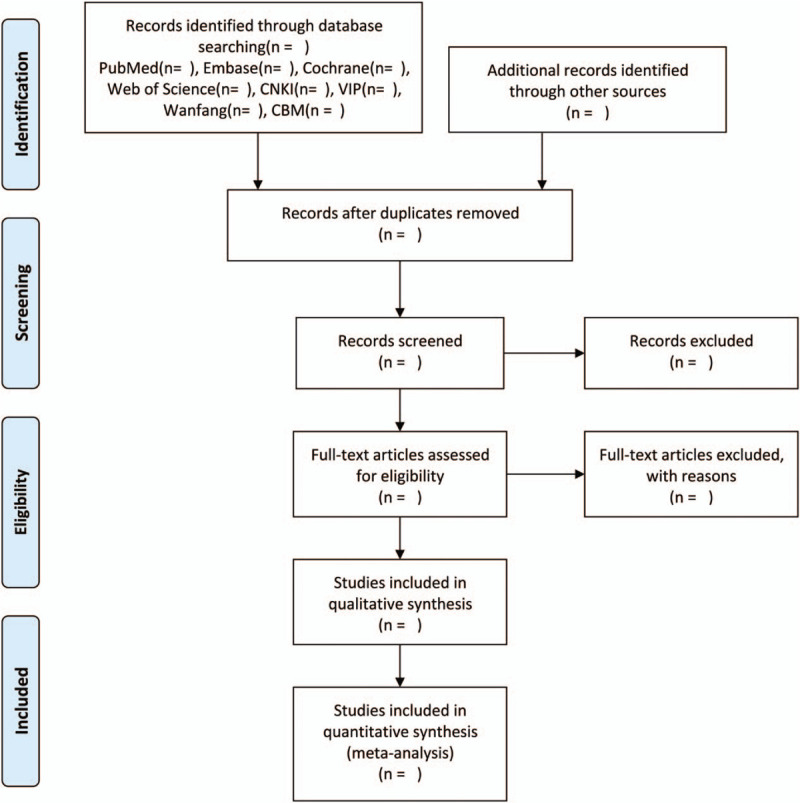
The PRISMA flow chart of the selection process. PRISMA = preferred reporting items for systematic reviews and meta-analyses.

We will use EndNote X9 to manage retrieved literature and eliminate duplicate data. Two investigators (LQC, CBT) will independently review the study titles and abstracts. Studies that fail to meet the required criteria will be excluded. Full texts will be evaluated and disagreements regarding study selection will be solved by discussion with a third reviewer (FXH). The reasons for exclusion will be documented. All selection procedures will conform to PRISMA guidance as outlined in (Fig. 1).

### 2.5 Data extraction and management

Data will be extracted by LQC and CBT using the standardized data extraction form based on the Cochrane Handbook.^[28]^ The standardized form including: Administrative information (author name, year of publication, country); Details of study (study design, recruitment, eligibility criteria, randomization, concealment, blinding, interventions and controls, sample size of both groups, follow-up term, type of Computer navigation system); Details of participants and procedural details: (mean screws per patient, deformity yes/no, minimally invasive yes/no, age, gender, number and specific vertebral levels instrumented with PS); all outcome indicators, and adverse events. If required, authors will be contacted for further information. Data extraction procedures will be assessed by a third reviewer (CRG).

### 2.6 Quality assessments

LQC and CBT will perform quality assessments and review the risk of bias using the Cochrane Collaboration's risk-of-bias assessment method (v6).^[29]^ This scale includes 7 risk of bias items, and each will be described as low, unclear, or high risk. Data will be presented in the risk of bias graph. Discrepancies will be resolved or as required, by a third reviewer (YY).

### 2.7 Strategy for data synthesis, assessment of heterogeneity

Data analysis and processing will be performed using STATA 16.0 (StataCorp, College Station, TX) by LQC and CBT. The Cochran *Q* and *I*^2^ statistic will be used for the assessment of statistical heterogeneity. Homogenous data will be compared using the Fixed-effects model (*Q* test with *P* >.1 and *I*^2^ <50%). Heterogeneous data will be compared using the random-effects (*Q* test with *P* < .1 or *I*^2^ statistic >50%). Standardized mean differences and 95% confidence intervals for continuous outcomes and relative risk for dichotomous outcomes with 95% confidence intervals will be used for the assessment of overall effect sizes. Qualitative and quantitative studies will be analysed separately and conclusions drawn. If quantitative synthesis is not appropriate, A systematic narrative synthesis will be generated in the form of text and tables.

### 2.8 Other analysis

#### 2.8.1 Subgroup assessments

These will be conducted based on minimally invasive versus open surgery, deformity surgery (5 or more spinal levels) versus no deformity surgery, lumbar versus thoracic Surgery.

#### 2.8.2 Sensitivity

Studies with a high risk of bias will be excluded to permit the evaluation of the robustness and reliability of the analysis.

#### 2.8.3 Publication bias

Funnel plots will be constructed and Egger regression tests will be performed for the assessment of bias for studies containing ≥10 RCTs. If publication bias is encountered, the fill and trim method will be used for subsequent analysis.

### 2.9 Evidence grading.

Grading of Recommendations Assessment, Development, and Evaluation will be used for the assessment of the quality of the evidence presented.^[30]^ Study limitations, inaccuracies, inconsistencies, indirect evidence, and publication bias will be used to rate evidence quality using 4 levels, namely:

(1) high;
(2) moderate;
(3) low; and
(4) very low.

### 2.10 Ethics and dissemination

All data will be disseminated through publications in peer reviewed scientific journals. The study includes no primary patient data or patient identifiers meaning local ethical approval is not required.

## 3 Discussion

Minimally invasive and digitization are the inevitable trend of spine surgery development. For improvements in the safety and accuracy of the procedures and to concomitantly reduce radiation exposure, navigation and robot technology are applied to the PS implantation, and continuously evolving. Navigation assisted PS implantation has a history of more than 20 years, and has been reported by many authors. But there is still no meta-analysis based on RCT about it. Therefore, our research will provide surgeons with evidence-based results on whether the navigation technology can achieve the purpose of safety, accuracy and reducing radiation exposure; meanwhile, we will evaluate the advantages and disadvantages of current navigation technology, and provide relevant support for the improvement or research and development of new navigation system and robot system.

## Author contributions

**Conceptualization:** Qianchun Li, Botao Chen, Xiaohong Fan

**Formal analysis:** Qianchun Li, Botao Chen, Xiaohong Fan

**Funding acquisition:** Xiaohong Fan

**Investigation:** Qianchun Li, Botao Chen, Yang Yu

**Methodology:** Qianchun Li, Leiming Jiang, Xiaohong Fan

**Supervision:** Yang Yu, Rigao Chen, Xiaohong Fan

**Validation:** Yang Yu, Rigao Chen, Xiaohong Fan

**Visualization:** QianChun Li, Botao Chen

**Writing – original draft:** Qianchun Li, Botao Chen, Rigao Chen, Yang Yu, Leiming Jiang, Xiaohong Fan

**Writing – review & editing:** Qianchun Li, Botao Chen, Rigao Chen, Yang Yu, Leiming Jiang, Xiaohong Fan
